# Pancreas grafts for transplantation from donors with hypertension: an analysis of the scientific registry of transplant recipients database

**DOI:** 10.1186/s12876-018-0865-0

**Published:** 2018-09-19

**Authors:** Zhen-Hua Hu, Yang-Jun Gu, Wen-Qi Qiu, Jie Xiang, Zhi-Wei Li, Jie Zhou, Shu-Sen Zheng

**Affiliations:** 1 Division of Hepatobiliary and Pancreatic Surgery, Department of Surgery, First Affiliated Hospital, School of Medicine, Zhejiang University, 79N, Qingchun RD, Hangzhou, China; 2 Zhejiang University School of Medicine, Hangzhou, Zhejiang China

**Keywords:** Pancreas transplantation, Hypertension, Type of transplant, BMI

## Abstract

**Background:**

With the rising demands for pancreas transplantation, surgeons are trying to extend the donors pool and set up a more appropriate assessment system. We aim to evaluate the effect of donor hypertension on recipient overall and graft survival rates.

**Methods:**

Twenty-four thousand one hundred ninety-two pancreas transplantation patients from the Scientific Registry of Transplant Recipients database were subdivided into hypertension group (HTN, *n* = 1531) and non-hypertension group (non-HTN, *n* = 22,661) according to the hypertension status of donors. Recipient overall and graft survival were analyzed and compared by log rank test, and hazard ratios of predictors were estimated using Cox proportional hazard models.

**Results:**

Patient overall and graft survival of non-HTN group were higher than that of the HTN group (both *p* < 0.001). The duration of hypertension negatively influenced both overall and graft survival rates (both *p* < 0.001). Multivariate analyses demonstrated that hypertension was an independent factor for reduced survival (hazard ratio [HR], 1.10; 95% confidence interval [CI], 1.01–1.18; *p* < 0.001). Other independent factors included recipient body mass index (HR, 1.02; 95% CI, 1.01–1.05; *p* < 0.001) and transplant type (pancreas after kidney transplants / pancreas transplant alone vs. simultaneous pancreas-kidney transplants; HR, 1.41; 95% CI, 134–1.55; *p* < 0.001).

**Conclusions:**

Donor hypertension is an independent factor for recipient survival after pancreas transplantation and could be considered in donor selection as well as post-transplant surveillance in clinical practice.

## Background

Pancreas transplantation is the main method to reestablish insulin secretion, and is reliable and repeatable for type 1 diabetes while less common for type 2 diabetes [1, 2]. It has been widely accepted that pancreas transplantation is an alternative to continued insulin therapy in imminent or established end-stage renal disease diabetic patients, who need combined kidney and pancreas transplantation to improve survival. As of December 2014, the International Pancreas Transplant Registry has recorded more than 48,000 pancreas transplantations [3]. The World Health Organization estimates that 9% of the global population is diabetic, and approximately 10% of this population have type 1 diabetes [1]. Even though minimally invasive approaches, such as islet transplantation, are being developed, pancreas transplantation remains the gold standard endocrine replacement treatment for complicated diabetes patients who cannot be optimally managed with conventional insulin therapy [2].

The pancreas transplantation survival rate has increased with developing techniques. Simultaneous pancreas–kidney transplants (SPK) has the best patient and graft survival, 96% and 83% for 1- and 5-year patient survival, 86% pancreas and 93% kidney graft function at 1st year. For pancreas after kidney transplants (PAK), at the first year, pancreatic graft function reaches 80%, while in pancreas transplant alone (PTA), pancreas graft function is 78% [1, 4, 5].

With the significant improvements of life expectancy and increasing demand for a higher quality of life, the demands for donor organs have been increasing steadily. Surgeons are trying to extend the donors pool and set up a more appropriate assessment system. Many donor and recipient characteristics have been discussed for qualified donor selection, recipient arrangement and post-transplant surveillance, including the type of transplantation, age of donors, HLA-mismatch and drainage, as well as the effect of hypertension, etc. Previous studies have demonstrated that the history and duration of donor hypertension was an independent factor for deceased donor kidney transplantation [6]. However, few studies have assessed the role of donor hypertension in pancreas transplantation. In this study, with analyses of the long-term follow-up data from Scientific Registry of Transplant Recipients, we aim to evaluate the effect of donor hypertension on recipient overall and graft survival in pancreas transplantation.

## Methods

We obtained data of 24,192 patients from the Scientific Registry of Transplant Recipients (SRTR). SRTR comprises data on all donors, waiting-list candidates, and transplant recipients in the USA, and is submitted by members of the Organ Procurement and Transplantation Network (OPTN). Patients were subdivided into groups that received pancreases from donors with (HTN) or without (non-HTN) hypertension. The hypertension history of donors was defined for the systolic blood pressure more than 140 mmHg or the diastolic blood pressure higher than 90 mmHg. And the duration of hypertension was calculated before the transplants. The characteristics of recipients and donors are showed in Table 1.

**Table 1:** Comparison of the baseline characteristics of recipients and donors

	HTN donors (*n* = 1531)	Non-HTN donors (*n* = 22,661)	P
Recipient characteristic			
Age (years)	40.0 ± 8.5	40.7 ± 8.3	0.342
Male	887 (58.0)	13,053 (57.6)	0.028
Ethnicity			
White	1243 (81.2)	18,540 (81.8)	0.515
Black	171 (11.2)	2447 (10.8)	0.521
Asian	12 (0.8)	249 (1.1)	0.643
Hispanic	102 (6.7)	1314 (5.8)	0.132
Other	3 (0.2)	111 (0.5)	0.897
Transplant before 1998	423 (27.6)	6481 (28.6)	0.078
BMI	24.8 ± 4.3	24.3 ± 4.9	0.021
Type of transplant			
PKA	161 (10.5)	3626 (16.0)	0.789
PAK	133 (8.7)	1994 (8.8)	0.754
SPK	1185 (77.4)	16,202 (71.5)	0.101
Other	52 (3.4)	839 (3.7)	0.119
Exocrine drainage			
Bladder drainage	531 (34.7)	8113 (35.8)	0.087
Enteric drainage	942 (61.5)	13,687 (60.4)	0.182
Others	58 (3.8)	861 (3.8)	0.763
Endocrine drainage			
Systemic system	1281 (83.7)	18,854 (83.2)	0.292
Portal system	231 (15.1)	3467 (15.3)	0.689
Other	19 (1.2)	340 (1.5)	0.037
HLA mismatch > 2/6	1408 (92.0)	20,372 (89.9)	< 0.001
PRA% > 20%	142 (9.3)	286 (18.7)	0.003
Years since DM onset	422 (27.6)	438 (28.6)	< 0.001
Follow-up in years	8.3 ± 5.6	8.3 ± 5.8	< 0.001
Donor characteristic			
Age (years)	54.2 ± 12.4	35.2 ± 16.0	< 0.001
Male	788 (51.5)	14,480 (63.9)	< 0.001
Ethnicity			
White	966 (63.1)	16,021 (70.7)	< 0.001
Black	344 (22.5)	2787 (12.3)	< 0.001
Asian	40 (2.6)	453 (2.0)	0.004
Hispanic	170 (11.1)	3195 (14.1)	0.011
Other	12 (0.8)	203 (0.9)	0.923
BMI (kg/m^2^)	27 ± 4	26 ± 5	< 0.001
Cause of death			
Anoxia	188 (12.3)	3784 (16.7)	< 0.001
Cerebrovascular accident	1131 (73.9)	6368 (28.1)	< 0.001
Head trauma	183 (12.0)	11,874 (52.4)	< 0.001
Other	28 (1.8)	634 (2.8)	0.003
Serum creatinine (mg/dL)	1.53 ± 1.21	1.44 ± 1.25	< 0.001
Cardiac arrest	58 (3.79)	770 (3.40)	0.024
WIT (min)	41.9 ± 19.0	41.5 ± 18.9	0.091
CIT (h)	7.5 ± 3.7	7.4 ± 3.5	< 0.001

### Recipients

Recipient variables included age (years), sex, ethnicity, year of transplant, body mass index (BMI), type of transplant, exocrine and endocrine drainage, human leukocyte antigen (HLA) mismatch, panel-reactive antibody (PRA), time since onset of diabetes mellitus (DM) to the surgery date, and date of final follow-up. Ethnicity was classified as White, Black, Asian, Hispanic, and Other. Type of transplant was categorized as PTA, PAK, and SPK. Exocrine drainage was divided into bladder drainage, enteric drainage, and others. Endocrine drainage was grouped into systemic system, portal system, and others.

### Donors

Donor characteristics were also compared between groups, including age, sex, ethnicity, BMI, cause of death, serum creatinine, cardiac arrest, warm ischemia time (WIT) and cold ischemia time (CIT). Cause of donor death was classified as anoxia, cerebrovascular accident, head trauma, and others.

### Statistical analysis

Continuous and categorical variables were compared using Student’s *t-*test and the chi-square test, respectively. The results were given as means ± standard deviation unless otherwise indicated. An alpha level of 0.05 indicated statistical significance. Pairwise deletion was used to handle missing data in each variable. Kaplan-Meier method was used to compare the overall and graft survival. Log-rank tests and multivariate Cox proportional hazard regression analyses were performed to obtain survival curves and for multivariate analyses. Univariate and multivariate Cox proportional hazards regressions of the entire cohort were performed to identify the predictors. A *p* value < 0.05 was considered statistically significant in univariate analysis as showed in Table 1, and were further selected for the multivariate model. All statistical analyses were performed using SPSS 20.0 (Armonk, NY: IBM Corp).

## Results

In our study, we obtained data on 24,192 patients, who were subdivided into HTN (*n* = 1531) and non-HTN (*n* = 22,661) groups.

Among the characteristics of recipients in the two groups, no significant differences were observed in age, ethnicity, transplant before 1998, exocrine and endocrine drainage (*p* > 0.05). HLA-mismatch, male and higher BMI were more frequent in HTN group. While in non-HTN group, PRA% > 20 is more common, and length of follow-up and duration of DM were slightly longer.

Donors in HTN group were much older than those in non-HTN group, and there were also fewer males in HTN group. There were more Black and Asian donors in HTN group, fewer White and Hispanic donors, while no differences in other ethnicity. Donors of HTN group had a higher BMI and longer CIT than those of non-HTN group, while there was no significant difference with regard to WIT. Serum creatinine was higher in HTN group than non-HTN group. A larger proportion of donors of HTN group died due to cerebrovascular accidents and cardiac arrest, with a smaller proportion from anoxia and head trauma, compared to the non-HTN.

We further analyzed the causes of graft loss and recipient mortality, which showed in Table 2. There were 1437 patients (103 for HTN and 1334 for non-HTN) went through the analysis of graft loss, and 1396 patients (99 for HTN and 1297 for non-HTN) went through the analysis of recipient mortality. In non-HTN group, graft loss due to vascular thrombosis and recipient death (with graft still function) were more frequent than HTN group, while HTN group had higher rate of anastomotic leak and pancreatitis. For recipient mortality, recipients died of cardiovascular in non-HTN group, were more frequent than HTN group, while recipients in HTN group died of other reason were more common than non-HTN group.

**Table 2:** Causes of graft loss and patient mortality

Cause	Graft loss			Cause	Recipient mortality		
	HTN	Non-HTN	*p* value		HTN	Non-HTN	*p* value
	(*n* = 103)	(*n* = 1334)			(*n* = 99)	(*n* = 1297)	
Hemorrhage	6 (0.058)	63 (0.047)	0.056	Graft failure	11 (0.111)	152 (0.117)	0.658
Vascular thrombosis	11 (0.107)	151 (0.113)	0.001	Cardiovascular	23 (0.232)	389 (0.300)	0.004
Anastomotic Leak	4 (0.039)	30 (0.022)	0.023	Organ failure	6 (0.061)	67 (0.052)	0.089
Rejection	2 (0.019)	23 (0.017)	0.106	Infection	31 (0.313)	441 (0.340)	0.308
Pancreatitis	5 (0.049)	61 (0.046)	0.021	Hemorrhage	8 (0.081)	98 (0.076)	0.112
Recipient death (graft still functioning at the time of death)	63 (0.612)	858 (0.643)	0.005	Other	15 (0.152)	38 (0.030)	0.001
Unknown	12 (0.117)	148 (0.111)	0.231	Unknown	5 (0.051)	112 (0.086)	0.793

**Fig. 1:**
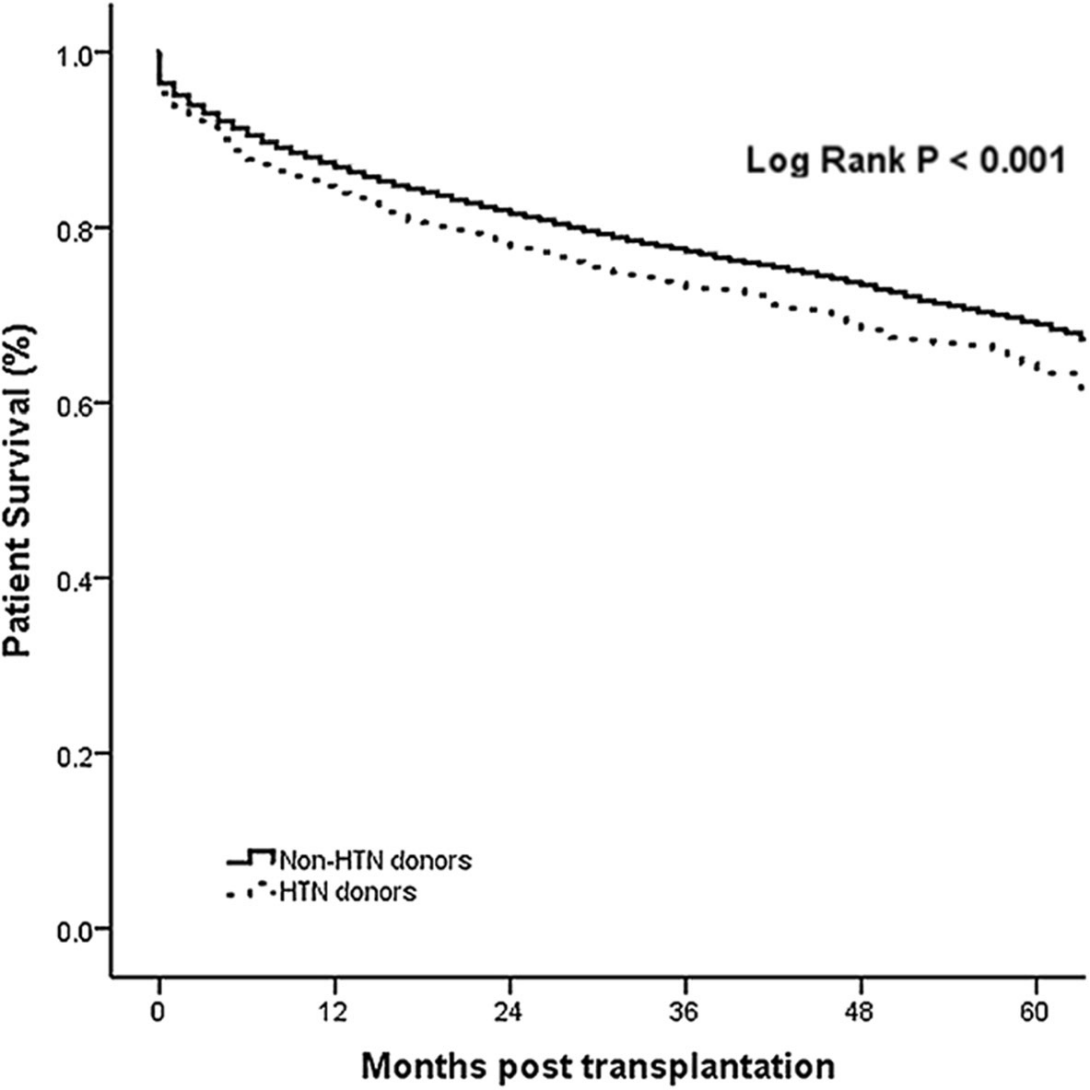
The comparison of overall survival for the Non-HTN and HTN groups

**Fig. 2:**
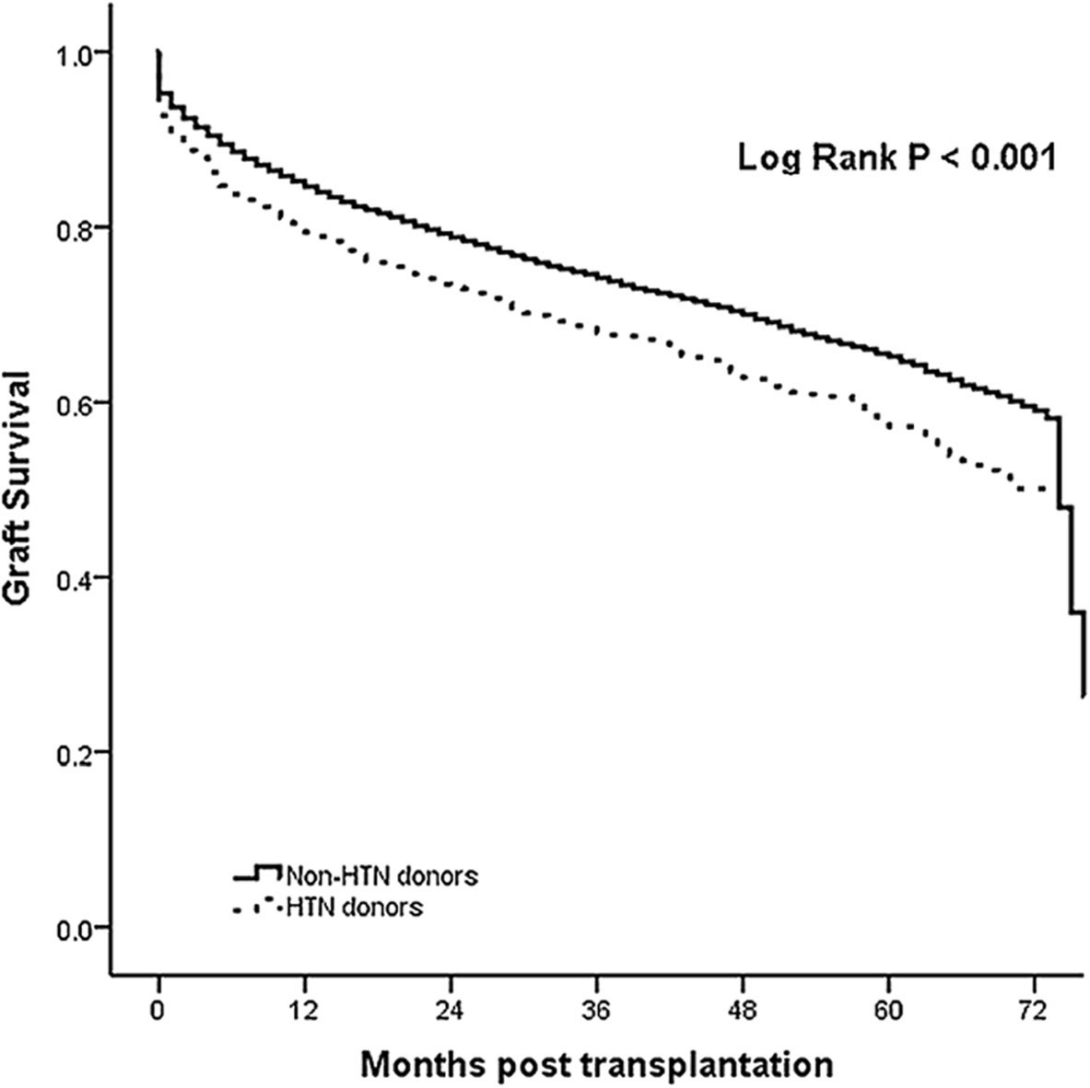
The comparison of graft survival for the Non-HTN and HTN groups

We followed up recipients for 5 years with the median follow-up period of 35 months. There were significant differences between recipients who received pancreases from non-HTN and HTN donors. The overall survival rate was significantly higher in patients who received pancreases from non-HTN donors (*p* < 0.001, Fig. 1), and the difference raised with time. Similarly, there was significant difference in graft survival between non-HTN and HTN donors (*p* < 0.001, Fig. 2).

**Fig. 3:**
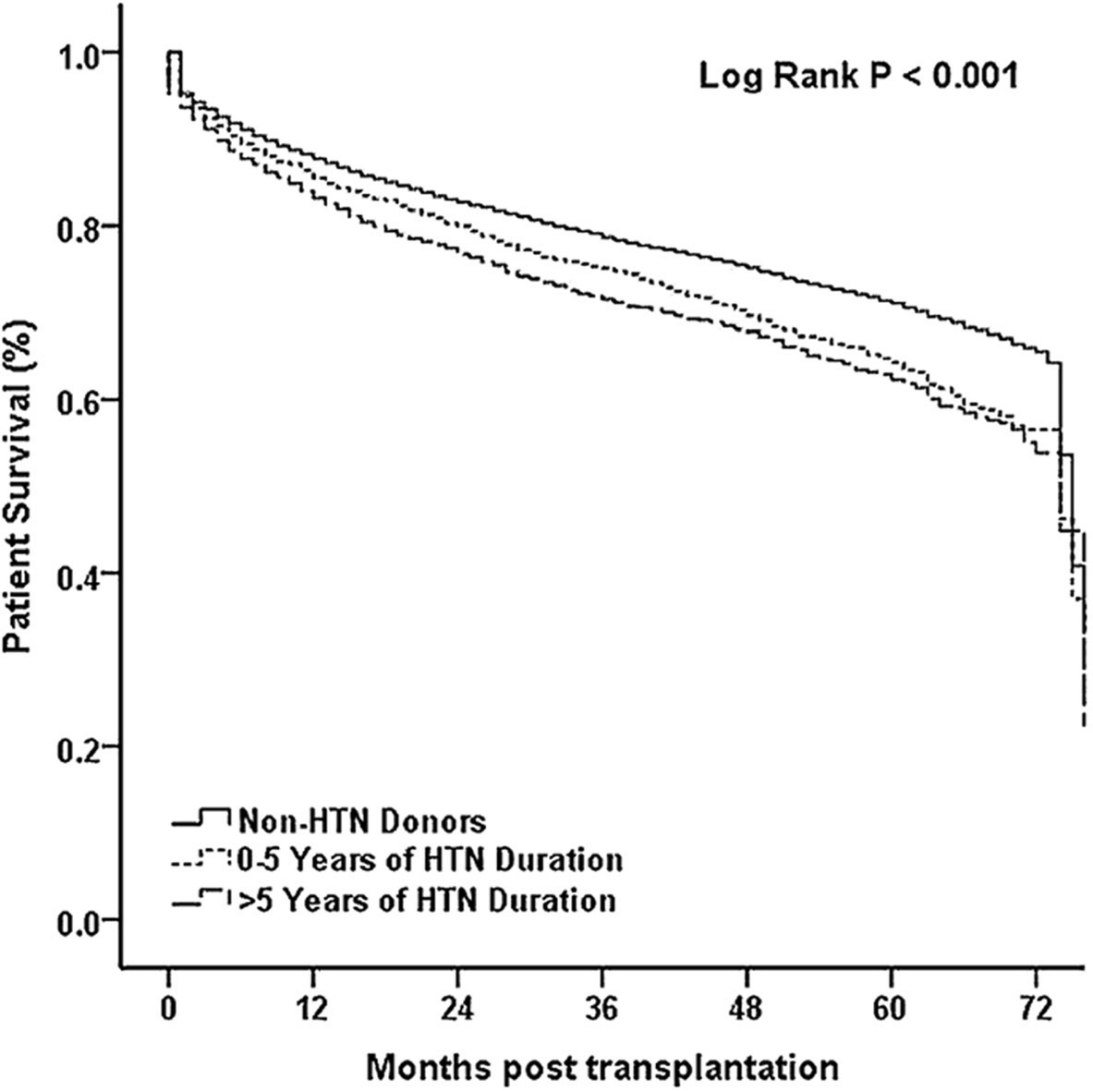
The comparison of overall survival for the HTN (0–5 vs. > 5 years of duration) and Non-HTN groups

**Fig. 4:**
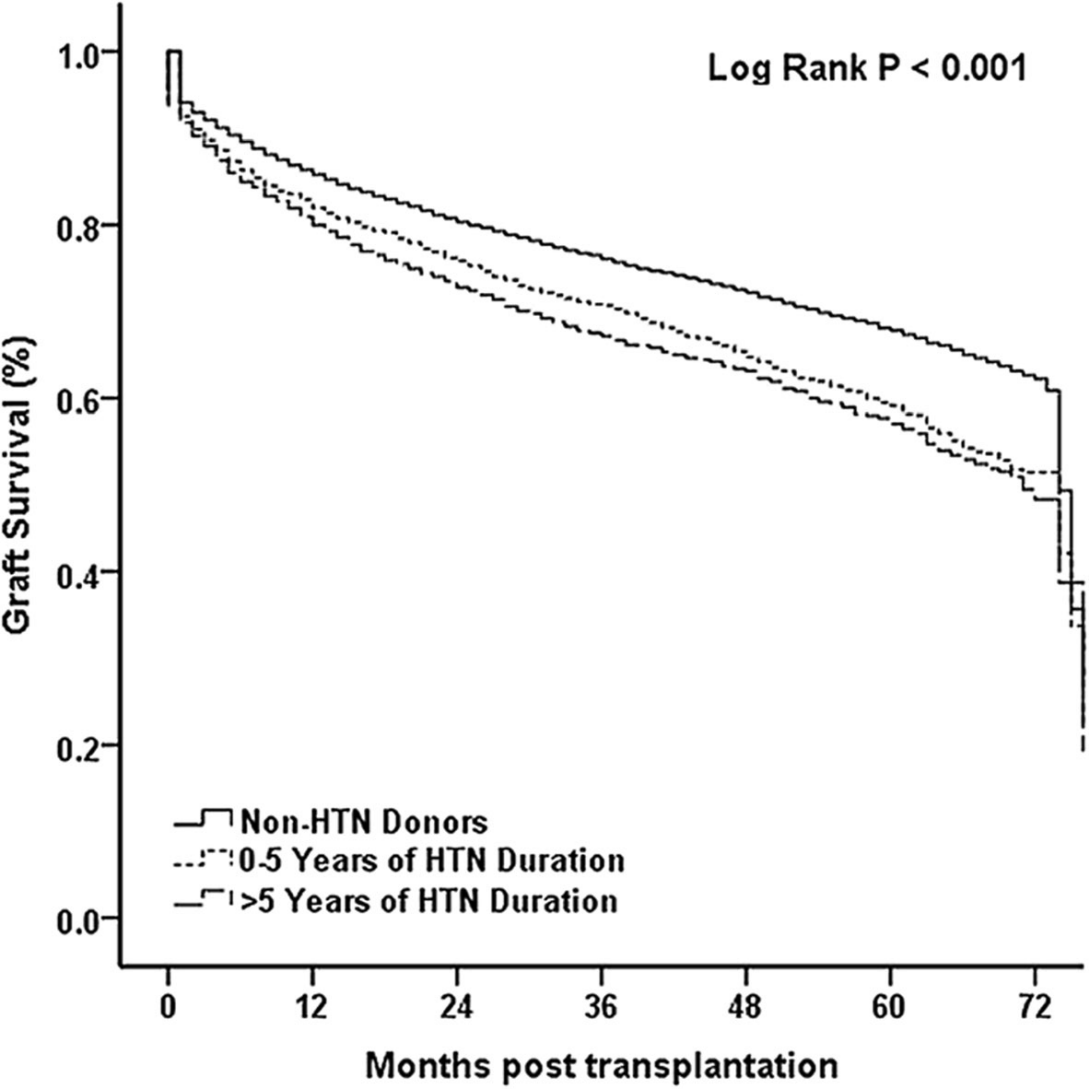
The comparison of graft survival for the HTN (0–5 vs. > 5 years of duration) and Non-HTN groups

We also evaluated the effect of hypertension duration on survival by dividing HTN group into 0–5 years and > 5 years durations. The longer duration of HTN, the lower recipient overall survival (*p* < 0.001, Fig. 3). Graft survival also significantly decreased with the lengthening HTN duration among non-HTN donors, HTN duration 0–5 years, and HTN duration > 5 years (*p* < 0.001, Fig. 4). However, no difference was observed among these 3 groups using Cox regression analysis (*p* > 0.05).

We analyzed recipient and donor characteristics to examine the independent effects of various factors. All the donors and recipients’ variables were accessed, including hypertension, age, sex, BMI, type of transplant and the other significant variables showed in Table 1.

We then performed the multivariate analyses and found that donor hypertension (HR, 1.10; 95% CI, 1.01–1.18; *p* < 0.001), recipient BMI (HR, 1.02; 95% CI, 1.01–1.05; *p* < 0.001) and transplant type (i.e., SPK; HR, 1.41; 95% CI, 134–1.55; *p* < 0.001) were independent predictors for both recipient overall and graft survival, showed in Table 3.

**Table 3:** Cox proportional hazard regression analyses

Variable	Univariate		Multivariate	
	HR	95% CI	HR	95% CI
HTN donors (ref: non-HTN donors)	1.34	1.17–1.45	1.10	1.01–1.18
Recipient BMI	1.03	1.01–1.06	1.02	1.01–1.05
Transplant type (PAK/PTA vs. SPK)	1.68	1.58–1.75	1.41	1.34–1.55

## Discussion

Our analyses indicated that hypertension of donor was an independent predictor of recipient overall survival (HR, 1.10; 95% CI, 1.01–1.18; *p* < 0.001). The longer duration of hypertension led to worse consequence. It has been confirmed that hypertension led to lower survival in kidney transplantation [6]. The hypertension of donor is negatively correlated with renal function in 6 months after transplantation [7]. However, the effects of hypertension seem to decrease with increasing age. When it comes to the transplanted kidneys from older donors (≥50 years old), graft survival is not so closely related to the history of hypertension until more than 10 years duration [8].

Solid organs from hypertensive donors are associated with lower recipient survival, which might be due to the damage of small vessels. The connection between kidney disease and hypertension is always considered as a villain–victim relationship because of the potential two-way causality [9].

Hypertension causes chronic pathological vascular changes, which ends to the microvascular modifications including internal elastic lamina reduplication of the arcuate and interlobar arteries, hyalinosis of the preglomerular vessel walls, and thickening of the intima. These result in fragile vessels, especially the small ones, which may lead to a poorer blood supply and higher sensitivity to microenvironment changes. In the kidney, these changes may lead to glomerular damage, glomerulosclerosis, patchy tubular atrophy, and interstitial fibrosis, which ultimately progress to nephrosclerosis, a form of intrarenal renovascular disease [9]. Hypertension might have similar effects on pancreas. The chronic pathological vascular changes leave a hypoxic condition in pancreas. Poorer oxygen reserve makes pancreas more sensitive to anoxia. The anoxia during the surgery is always companied by a higher rate of post-transplant complications, like focal necrosis of the pancreas, secondary pancreatitis, pancreatic leakage and delayed graft function, etc.

Poorer blood supply not only causes pathological changes in the organs but also affects graft survival. It’s hypothesized that there is a higher death rate of kidney or pancreas cells in the oxygen-poor postoperative environment [10, 11]. The dead cells activate the immune system, which can lead to autoimmune problems [12, 13]. The higher organ cell death rate, the more postoperative complications and graft rejection. Following the acute or chronic rejection, immune-suppressor may go through the worse. T-cell depletion antibodies in association with maintenance combination, including calcineurin inhibitors (CNI), antimetabolites, like mycophenolate mofetil (MMF), and corticosteroids (Cs), were recommend for prevent allo- and auto-immune reactions [14]. However, CNIs have nephrotoxic and especially diabetogenicity, MMF causes gastrointestinal pathological changes and leukocytopenia, Cs induce hyperinsulinemia, hyperinsulinism and insulin resistance. The extra immune-suppressor usage finally increases the complications and exacerbates the graft function.

The microvascular modifications, poor blood supply, acute or chronic rejection and extra immune-suppressor, lead to fatigue pancreas. Further, the early pathological changes of organs, like mild hypertensive nephropathy and focal pancreatic infarcts, could not been identified appropriately before transplants. In this study, the variable, death by CVA, was significant in univariate analysis, while there was no difference in the multivariate analysis. Thus, besides vasculature, other pathophysiological processes of HTN also contribute to the disfunction of donor pancreas. These factors can be enhanced by the duration and bad control of hypertension, finally resulting in the lower patient and graft survival rates.

SPK has been recommended for pancreas transplantation by most surgeons because of its higher survival (73% for 5- and 56% for 10- year pancreas graft function of SPK vs 64% and 38% of PAK vs 53% and 36% for PTA) [15]. Though SPK has the highest technical failure (TF) failure (15.3% vs PAK 12.2% and PTA 11.4%) [16, 17], it has the lowest graft failure rate (30.6%). This probably dues to its lower chronic rejection rate (3.7% vs PTA 11.3% and PAK 11.6%) [18, 19], and immune avoid of kidney graft (immunologic protection exerted by the kidney [20]; easier reject diagnosis by serum creatinine and histology of kidney [18, 20]).

The influence of the type of transplantation is also related to the patient’s condition. SPK and PAK are performed for diabetic kidney recipients, while PTA is appropriate for non-uremic labile diabetics [21]. SPK is performed mostly in patients with insulin-dependent DM and dialysis-required chronic renal failure. These patients have better insulin control, which means better insulin response of the peripheral organs, and reestablishing internal secretion would greatly improve retina, vascular, and nerve functions. In PAK, the kidney usually comes from a living donor, while only 0.5% of pancreatic transplantations are from living donors [22]. PTA is usually performed in patients with poorly controlled insulin-dependent DM but with stable renal function. However, 30% of these people will eventually need a renal transplantation [23], which means double surgical strike.

BMI is also an important predictor of survival, including both recipient and donor BMI. Obesity is accompanied by various complications during and after the operation [24], including percutaneous drainage, relaparotomy, delayed kidney graft function, acute rejection within the first post-transplant year, and vascular thrombosis of the pancreatic graft. According to our study, we supported that the overweight of recipients was associated with worse prognosis. Recent studies also showed that obesity was associated with higher patient death and kidney graft loss [25], which probably due to the higher rates of death and graft failure in the first 30 days [26].

On the other hand, overweight donors should be deliberative. Axelrod et al. set the Pancreas donor risk index (PDRI), and indicated that if the BMI raised from 24 to 30, the donor risk index (DRI) would increase to 1.17 [27]. Although some researchers have tried to extend the donor pool by overweight donors [28], obese donors are more likely to have a fatty pancreas with poor vascularization and more prone to ischemia–reperfusion injury and fat necrosis, which would finally lead to fluid collection and infected nidus [29]. Besides, obese donors pose a technical challenge in the preparation of their massive abdominal wall and fatty viscera. Furthermore, pancreases from obese donors are predisposed to peri-pancreatic fluid collection, which is associated with early graft pancreatitis [30]. Therefore, individuals with BMI > 35 kg/m^2^ are never recommended as donors [2].

The limitation of this research is that we had not estimated the control of hypertension, especially the vasoactive agent usage. Effective blood pressure management, including exercise, diet, psychology and vasoactive agent usage etc., can well prevent the organ damages caused by hypertension. More researches are needed to estimate the different effects of classificatory of hypertension. Besides, we only analyzed the hypertension duration for less than 5 years and more than 5 years. More classificatory researches could help confirm the effect of hypertension duration.

## Conclusion

In this study, we analyzed 24,192 patients from SRTR, and found that hypertension of donors had negative effects on transplant prognosis. These may due to the degenerated vessels net and changed microenvironment caused by hypertension. The evidence that a longer hypertension duration was associated with worse prognosis also demonstrated the organ damage by hypertension. It might be helpful to improve the vessels net of donor pancreas before transplant, including the vasodilators usage, diuretic or slight volume expansion, and adequate perioperative perfusion. Finally, more studies are required to evaluate the status of donor hypertension into evidence-based selection criteria in clinical practice.

## Abbreviations

BMI: Body mass index
CIT: Cold ischemia times
CNI: Calcineurin inhibitors
Cs: Corticosteroids
DM: Diabetes mellitus
HLA: Human leukocyte antigen
HTN: Hypertension
MMF: Mycophenolate mofetil
PAK: Pancreas after kidney transplants
PRA: Panel-reactive antibody
PTA: Pancreas transplant alone
SPK: Simultaneous pancreas–kidney transplants
SRTR: Scientific Registry of Transplant Recipients database
TF: Technical failure
WIT: Warm ischemia times
